# An Investigation of Current Endodontic Practice in Turkey

**DOI:** 10.1100/2012/565413

**Published:** 2012-12-02

**Authors:** R. F. Kaptan, F. Haznedaroglu, M. B. Kayahan, F. B. Basturk

**Affiliations:** ^1^Department of Endodontics, Faculty of Dentistry, Yeditepe University, 34755 Istanbul, Turkey; ^2^Department of Endodontics, Faculty of Dentistry, Istanbul University, 34116 Istanbul, Turkey; ^3^Department of Endodontics, Faculty of Dentistry, Marmara University, 34722 Istanbul, Turkey

## Abstract

*Objectives*. The aim of this study was to gather information about the quality and quantity of root canal treatments carried out by general dental practitioners in Turkey. *Methods*. Questionnaires were given to 1400 dentists who attended the 16th National Congress organized by the Turkish Dental Association. The participants were asked to answer 34 multiple-choice questions. The questions were subdivided into 3 main topics; general information; general approach to endodontic treatment; and cleaning, shaping, and obturation of root canals. The statistical analysis was carried out by an *χ* ^2^-test to compare the means at a significance level of *P* < 0.05. *Results*. The response rate for this study was 43%. There was a wide variation in the number of root canal treatments completed per month. Nearly 92% of practitioners stated that they never used rubber dam. The most commonly used working length determination technique was radiographic evaluation (*P* < 0.05). Sodium hypochlorite was the irrigant of choice with varying concentrations and AH Plus was the sealer of choice (*P* < 0.05). Resin composite was the most frequently used material for final restorations. *Conclusions*. Endodontic procedures in general practice in Turkey have differences from widely acknowledged quality guidelines. Despite the introduction of new instruments and techniques, most of the general practitioners chose conventional methods.

## 1. Introduction

The attitudes and approaches of general dental practitioners (GDPs) toward endodontic therapy reflect the quality of the root canal treatment (RCT) conducted in a country. Success in endodontic treatment depends on adequate preparation of the root canal space and obturation of the root canal system to prevent the passage of microorganisms and fluid along the root canal [1]. 

In 2001 the European Society of Endodontology (ESE) published undergraduate curriculum guidelines for endodontology [2] which aimed to standardize the quality and quantity of education and clinical experience received during undergraduate training in dental schools in Europe. To improve the standards in clinical practice, undergraduate training in endodontics should be undertaken in a way that a minimum level of competence as well as an ethos of continued learning is instilled in the graduate [2]. Standardized undergraduate endodontic training is important for preventing disparities in endodontic treatment carried out by general dental practitioners from different universities in a country or even different countries.

The purpose of this research is to gather information about the quality and quantity of root canal treatments carried out by general dental practitioners in Turkey.

## 2. Methods

Questionnaires were given to 1400 dentists who attended the 16th National Congress organized by the Turkish Dental Association (TDB). Six hundred and two dentists returned the questionnaires. Respondents were not asked for any identification in order to guarantee anonymity. The participants were asked to answer 34 multiple-choice questions. The questions were subdivided into 3 main topics; general information; general approach to endodontic treatment; and cleaning, shaping and obturation of root canals.

1. General information: Gender, years of professional activity, working situation.
2. General approach to endodontic treatment: Frequency of root canal treatment, use of rubber dam, use of magnification, and frequency of treatment of a fourth canal in a maxillary molar.
3. Cleaning, shaping and obturation of root canals: choice of preparation technique and instrument, method of determination of the working length, preference of root canal irrigants, concentration of sodium hypochlorite, use of side-perforated needles, root canal obturation technique, choice of sealer, temporary filling material used, and choice of final restoration.

In order to make a more detailed comparison of the data, the sample was divided according to years of professional practice, as follows: up to 5 years; 6–10 years; 11–15 years, 16–20 years and more than 20 years.

The data was checked and entered in a personal computer and analysed using Number Cruncher Statistical System software 2007 (NCSS, Utah USA). The statistical analysis was carried out by an *χ* ^2^-test to compare the means at a significance level of *P* < 0.05.

## 3. Results

### 3.1. Response Rate

The response rate for this study was 43%. From the 1400 questionnaires distributed, 602 replies were received of which 589 were deemed usable. A total of 74.6% of individuals who answered the questionnaire were either employed in dental schools or in public health care practice. The remainder of the sample were employed in private practice (Table 1). 

The rate of dental practitioners working in public health care with an experience of 0–5 years, 6–10 years, and 11–15 years in the profession was found to be statistically and significantly higher than the 16–20 years and >20 years groups (*P* = 0.0001). 

For years of professional activity, there appears an insignificant overrepresentation of practitioners working for less than 5 years. For these variables, we can accept that the distribution of the present sample is representative for the total Turkish dental practitioner population. 

Of all respondents, 52.1% were male. 

### 3.2. General Approach to Endodontic Treatment

There was a wide variation in the number of RCTs completed per month ranging from 1 to over 40 (mean: 12.8); there was no correlation between the number of RCTs and the age of the practitioner. Forty percent of respondents stated that they complete between 10 and 19 root canal fillings each month (Table 2).

Less than 2% of individuals stated that they always used rubber dam for endodontic treatment whereas 91.9% of practitioners replied that they never used rubber dam. There was no relationship between its use and years of professional practice (*P* = 0.076).

A fourth canal in a maxillary molar was prepared and filled in a minority of cases. Of all respondents, 11.6% reported that they never detect the canal and 58.3% stated they rarely detect and treat the canal. There was no correlation between detecting and treating the second mesiobuccal canal of a maxillary molar and years of professional practice (*P* = 0.397).

Of all respondents, 17.1% reported using loupes and only 3.2% reported using a dental operating microscope for endodontic treatment.

### 3.3. Cleaning, Shaping, and Obturation of Root Canals

There was a significant tendency among practitioners working for over 20 years toward using tactile sensation to estimate working length (24.10%). This percentage decreases significantly as the years of practice decreases (*P* < 0.008). Radiographic evaluation was the most commonly used method for working length determination (77.8%) followed by the use of an electronic apex locater (41.1%) with or without radiographs.

The standardized method of canal preparation was mostly chosen by older practitioners (*P* = 0.029), whereas the step-back technique was the main method of canal preparation for younger practitioners (*P* = 0.0001). Independent of years of dental practice, Ni-Ti rotary instruments were used by all of the practitioners (Table 3).

Preference of root-canal irrigants and concentration of sodium hypochlorite according to years of professional experience is shown in Table 4. Side-perforated needles were rarely used for irrigation in endodontic practice in Turkey. Only 10.5% of the respondents stated that they use side-perforated needles all the time whereas 69.5% reported that they never use them. Interestingly, side-perforated needles were mostly favoured by older practitioners (*P* = 0.0001).

Cavit (ESPE, Neus, Germany) was the most popular temporary filling material (75.3%), followed by zinc phospate cement (23.7%) and zinc oxide eugenol and IRM (Dentsply De Trey, Konstanz, Germany) (14.9%). Other unspecified materials were used by 9.2% of the respondents. Time since graduation had no statistically significant influence (*P* > 0.04) on the choice of temporary filling material.

As shown in Table 5, obturation of root canals using gutta-percha and sealer without lateral condensation was the method most favoured by general dental practitioners in Turkey, followed by the cold lateral condensation technique. Endomethasone (Spécialités Septodont, Saint Maur, des Fossés, France) and AH Plus Dentsply De Trey, Konstanz, Germany) were the most commonly used sealers for root canal filling. 

For the final restoration, resin composite was the most frequently used material (79.8%). Crown restoration was favoured by almost half of the respondents (49.5%) followed by amalgam filling (25.8%). Years of professional activity had no influence on the choice of final restoration (*P* > 0.1).

## 4. Discussion

The aim of this study was to gather information on the preference of choice of the materials, methods, and current trends employed in root canal treatments by Turkish dentists.

The response rate for this study was 43%. The questionnaires were anonymous, so no attempt was made to follow up on the questionnaires that were not returned. The response rate was disappointingly low compared to the survey of Hommez et al. [3] (99.4%). But in their study, the questionnaires were given and collected by the participants individually. In our study, the number of questions and participants who received questionnaires was relatively higher. The response rate of this questionnaire was found satisfactory compared to studies that used mail survey methods [4, 5], and postal survey methods [6]. The response rate of our study is relatively lower compared to some similar studies [7–9]. The reason for this difference may be due to the higher number of participants (*n* = 1400) and the number of questions (*n* = 34) in our survey.

To date, no study had been established to gather information on the RCTs carried out by dentists working in Turkey. Thus, it is not possible to compare the current treatment approach to earlier periods. The data collected in our investigation might be of value in providing information and baseline data for future investigation of changes in endodontic practice in Turkey.

The majority of respondents (84%) were general dental practitioners. This information reflects the fact that this is the sector in which the majority of dental treatment is provided in Turkey. Of all the respondents, 74.6% of the dental practitioners worked in private practices, whereas 25.9% worked in public health services (e.g., public hospitals, dental schools). Thus, the information obtained may be representative of the general dental population throughout Turkey. The ratio of public health practitioners working in hospitals for 15 years or less to those who have worked for 16 years or more was found to be significantly higher (*P* = 0.0001). During the last decade, governments have made significant changes in health policies. Hence, new graduates have chosen to work in public health services rather than private practices.

Of all the respondents, 98.3% of the practitioners perform RCTs on a daily basis. Compared to some developing countries, like Kenya (67%) [10], this rate was found to be significantly higher, whereas other developing countries like Sudan (95%) [8] had similar numbers. The respondents of our questionnaire mentioned their interest in endodontics and the referral of difficult cases to an endodontist was not common practice. This might be the reason for the higher percentage performing RCT. The percentage of those performing more than 10 RCTs a month was found to be 74.7%. Another important finding is that practitioners who have been working for 5 years or less perform significantly more RCTs than those working for more years (*P* = 0.002). Modern endodontic instruments seem to be more accessible than was previously the case and, in addition, the teaching of new techniques and materials in dental schools might be leading new graduates to prefer endodontic treatment, rather than extraction.

According to the ESE guidelines [1], RCT procedures should be carried out only when the tooth is isolated by rubber dam, on the basis of infection control and endodontic outcomes, as well as the dangers of practising without adequate oropharyngeal protection [11]. Even though the use of rubber dam is taught in every dental school and is mandatory for undergaduate students in Turkey, its use in daily dental practice is abandoned quickly after graduation. The reasons for not using rubber dam were that it is time consuming, not readily available, and expensive when available and that patients do not prefer its use. The percentage of practitioners who do not use rubber dam was found too be 91.9% regardless of the time after graduation. This finding was in accordance with other studies [3, 4, 7, 12]. 

The majority of dental practitioners in Turkey had difficulties finding the fourth canal in upper molars, even though it may be present in the majority of maxillary first and second molars [13]. This finding is similar to the findings of Hommez et al. [3] and Slaus and Bottenberg [4]. The low percentage of using loops and dental operating microscopes might explain why the fourth canals in upper molars were difficult to detect and treat.

The reliance on the preoperative radiograph and tactile sensation to determine working length cannot be recommended in modern endodontics, because the instruments may bind against the canal walls [14], or may perforate apically, causing underfilling or overfilling. Most of the practitioners who relied on tactile sense for estimation of working length have been working for over 20 years (24.10%). This percentage decreases significantly as the years of practice decrease (*P* < 0.008). Radiographic evaluation is the method favoured by the majority of respondents (77.8%). The use of an electronic apex locater to determine working length has gained in popularity and is being taught at the undergraduate level in Turkey. Even though 41.1% of all respondents use electronic apex locaters, they often do so in conjunction with radiographs. This finding is in accordance with Jenkins et al. [12] and Palmer et al. [7].

The standardized method of canal preparation, utilizing instruments of fixed size and taper, with the use of a single point for obturation [15], is commonly chosen by most of the older practitioners. Practitioners working for less than 5 years tended to choose the step-back technique. Even though the use of reciprocating devices has been taught in dental schools only for the last decade or less, this method was also well established amongst practitioners, independent of their years of dental practice. This finding is in contrast with the findings of Jenkins et al. [12] but in accordance with those of Hommez et al. [3]. Faster and simpler preparation of root canals might be the reason for general practitioners using rotary instruments so commonly.

Root canal systems are complex and no instrument or method for cleaning and shaping available to the clinician can entirely remove tissue remnants or debris smeared on the canal walls [16]. Thus, the use of an antimicrobial irrigant solution is needed to debride the accessory anatomy by chemical means [12]. In this study, sodium hypochlorite was the most popular amongst most of the practitioners (90.2), followed by EDTA (44.1%) and H_2_O_2_ (38.4%). Local anesthetic solution, which is commonly used in the UK [11, 12] is the least preferred irrigant. However, 1.2% of all the respondents reported that they did not use any irrigating solutions. Many clinicians prefer dilute concentrations to reduce the caustic effect of sodium hypochlorite on oral and periapical tissues [12]. The limited use of rubber dam may be a factor in the choice of more dilute solutions [4, 11]. An interesting finding is that 7% of the respondents, most of whom were older practitioners (*P* < 0.006), stated that they did not know the concentration of sodium hypochlorite they have been using. Also, interestingly, older practitioners were the ones who mostly favoured the use of side-perforated needles for irrigation (*P* = 0.0001). According to the GDPs in Turkey, side-perforated needles on the dental market are expensive and this might be the reason for its limited use.

In the present study, calcium hydroxide was used by 61.5% of the respondents, which is comparable to the 69.7% in Flanders (Belgium) [17] and the 63% in North Jordan [9], and considerably more than the 9% in the USA [18], the 7% [12] in the UK. These differences between countries may be attributed to the different preclinical teaching regime between universities [19]. The use of calcium hydroxide amongst practitioners working for over 20 years was found significantly less than the rest of the practitioners (*P* < 0.002). About 6% of practitioners stated they did not use any intracanal medication.

In endodontics, temporary restorative materials must provide a high-quality seal of the access preparation to prevent microbial contamination of the root canal [4]. Seventy-five percent of the respondents use Cavit as temporary filling material. Cavit has good sealing properties for up to three weeks when used in simple endodontic access cavities [20]. It has been marketed for over 50 years and has not been replaced by any new temporary restorative materials for sealing access cavities [5].

Like materials and methods for instrumentation, numerous methods of obturation are available to the clinician [16]. The root canal filling should consist of a (semi-) solid material with a sealer to fill the voids between the material and the root canal wall [1]. According to the survey of Qualtrough et al. [19], cold lateral condensation has been the most popular undergraduate root-filling technique. However, in our study, using gutta-percha with sealer without lateral condensation was favoured by most of the respondents (55.3%), followed by cold lateral condensation (33.8%). Using gutta-percha in conjunction with a sealer is a relatively simple and versatile technique that does not require expensive equipment [12]. This might be the reason why this technique is used by the majority of responding practitioners in their general practice. The finding for the cold lateral condensation method is relatively lower than the findings of Palmer et al. [7] (75%) and Al-Omari [9] (46.6%). While some dentists are using techniques taught during their undergraduate education, there are a number of dentists using techniques with no evidence of clinical effectiveness that they were not taught in their undergraduate course [7, 12]. Although single-point technique is not being taught nor recommended in dental schools, it has been used by 26.8% of all respondents. Its simplicity might be the reason for its frequent use. Similarly, paste-only root fillings are difficult to control with the risk of under- or overfilling of the canal [12], but 3.2% of respondents used only paste to obturate the root canal system. Though warm gutta-percha filling techniques are not taught in the majority of dental schools in Turkey, it's been used by 3.5% of the respondents of all age groups. This finding shows that a number of practitioners made an effort to use filling techniques other than those taught in undergraduate education.

The most popular root-canal sealer amongst Turkish GDPs was AH Plus, followed by Endomethasone. This finding is in accordance with the findings of Hommez et al. [17] and in contrast with the findings of Jenkins et al. [12], Ahmed et al. [8], and Al-Omari [9], who found that the majority of the respondents used zinc-oxide-based sealers. These differences are likely to be attributed to different materials and methods used in dental training between universities [19].

According to the ESE quality guidelines for endodontics [1], the tooth should be adequately restored to prevent bacterial recontamination of the root canal system or fracture of the tooth. In the present survey, adhesive restoration was the choice of final restoration and resin composite was the material of choice. The use of a crown or an inlay/onlay restoration was relatively lower compared to the findings of Palmer et al. [7]. Economic considerations might be the reason most of the practitioners choose relatively cheaper resin composite restorations instead of crown or inlay/onlay restorations. 

The decisions of general dental practitioners regarding treatment options and referrals when confronted with periapical lesions were also investigated. In cases featuring a periapical lesion, the preferred treatment approach is RCT. It is followed by apical resection in conjunction with RCT. The number of RCTs performed decreases with the increase of the size of the periapical lesion. This result is in accordance with the findings of Hommez et al. [17]. Referral of such cases to an endodontist was not common practice (0.8%). 

In conclusion, the results have shown that endodontic procedures in general practice in Turkey have differences from widely acknowledged quality guidelines. Despite the introduction of new instruments and techniques, most of the general practitioners chose conventional methods. Future investigation will be needed to assess changes in endodontic practice in Turkey. 

## Figures and Tables

**Table 1:** Working situation and gender according to years of professional experience.

Years of Professional activity		0–5 years	6–11 years	11–15 years	16–20 years	>20 years	*P*
Working situation	Private	(98) 57.0%	(81) 77.1%	(69) 75.8%	(71) 80.7%	(131) 89.1%	
	Public	(74) 43.0%	(24) 22.9%	(22) 24.2%	(17) 19.3%	(16) 10.9%	**0.0001**

Gender	Female	(80) 46.5%	(51) 51.4	(49) 53.8	(40) 45.5%	(66) 44.9%	
	Male	(92) 53.5%	(51) 48.6%	(42) 46.2%	(48) 54.5%	(81) 55.1%	0.611

**Table 2:** Frequency and number of endodontic treatments according to years of professional experience.

Years of Professional activity		0–5 years	6–11 years	11–15 years	16–20 years	>20 years	*P*
Number of root canal treatments per month	1–9	(27) 16.1%	(13) 13.1%	(22) 24.7%	(22) 26.8%	(55) 39.6%	
	10–19	(65) 38.7%	(43) 43.4%	(40) 44.9%	(34) 41.5%	(49) 35.3%	
	20–29	(31) 18.5%	(20) 20.2%	(11) 12.4%	(12) 14.6%	(18) 12.9%	
	30–39	(22) 13.1%	(12) 12.1%	(7) 7.9%	(5) 6.1%	(5) 3.6%	
	>40	(21) 12.5%	(10) 10.1%	(9) 10.1%	(7) 8.5%	(10) 7.2%	**0.002**

**Table 3:** Percentage of the choice of preparation technique and instrument, use of Ni-Ti rotary instrument according to years of professional experience.

Years of professional activity		0–5 years	6–11 years	11–15 years	16–20 years	>20 years	*P*
Choice of preparation technique	Step-back	58.9%	37.4%	36.0%	20.7%	16.4%	**0.0001**
	Step-down	20.2%	18.2%	29.2%	30.5%	29.3%	
	Ni-Ti rotary technique	33.3%	45.5%	43.8%	51.2%	45.7%	
	Conventional technique	18.5%	23.2%	27.0%	18.3%	32.9%	0.029

**Table 4:** Percentage of the preference of root canal irrigants and concentration of sodium hypochlorite according to years of professional experience.

Years of professional activity		0–5 years	6–11 years	11–15 years	16–20 years	>20 years	*P*
Choice of root canal irrigants	NaOCl	93.5%	92.0%	94.4%	95.1%	85.7%	
	Saline	49.7%	42.0%	37.1%	34.1%	27.9%	**0.002**
	EDTA	41.7%	42.0%	52.8%	57.3%	38.6%	**0.03**
	CHX	33.3%	39.0%	43.8%	43.9%	40.0%	
	H_2_O_2_	16.1%	18.0%	23.6%	20.7%	25.7%	
	Local anesthesia	3.0%	6.0%	3.3%	2.4%	2.1%	
	Do not use	1.2%	2.0%	1.1%	1.2%	0.7%	

Concentration of sodium hypochlorite	0.5%	11.3%	13.1%	11.2%	7.3%	12.9%	
	1%	1.8%	2.0%	6.7%	6.1%	7.1%	
	2.5%	53.0%	39.4%	47.2%	39.0%	41.4%	
	5%	25.0%	41.4%	25.8%	32.9%	20.7%	
	No idea	4.8%	1.0%	6.7%	9.8%	12.9%	
	Do not use	4.2%	3.0%	2.2%	4.9%	5.0%	

**Table 5:** Choice of obturation technique, sealer, temporary filling material, and permanent filling material according to the years in dental practice.

Years of Professional activity		0–5 years	6–10 years	11–15 years	16–20 years	>20 years	*P*
Obturation technique	Paste only	1.8%	2.0%	4.5%	2.4%	6.4%	
	Thermafil	3.0%	2.0%	5.6%	3.7%	3.6%	
	Cold lateral condensation	53.0%	43.4%	39.3%	12.2%	15.7%	0.000
	Single cone	20.2%	25.3%	21.3%	35.4%	34.3%	0.016
	Cone + sealer without lateral condensation	48.8%	52.5%	57.3%	64.6%	62.9%	0.056
	Vertical condensation	8.9%	9.1%	9.0%	6.1%	5.7%	

Choice of sealer	Endomethasone	25.0%	31.3%	28.1%	40.2%	40.7%	0.02
	Iodoform-based sealer	1.2%	2.0%	3.4%	1.2%	2.9%	
	AH Plus	54.2%	55.6%	52.8%	39.0%	39.3%	0.014
	Diaket	12.5%	14.1%	13.5%	15.9%	10.7%	
	Sealapex	10.1%	5.1%	18.0%	19.5%	12.1%	
	AH26	32.7%	24.2%	27.0%	19.5%	20.0%	
	Roekoseal	6.0%	3.0%	2.2%	3.7%	4.3%	
	Other	1.8%	1.0%	0.0%	3.7%	9.3%	

Temporary filling material	Cavit	79.2%	74.7%	76.4%	81.7%	66.4%	0.057
	Phospate cement	23.2%	26.3%	19.1%	23.2%	25.7%	
	ZnOE + IRM	17.3%	13.1%	22.5%	13.4%	9.3%	0.071
	Other	6.0%	7.1%	15.7%	6.1%	12.1%	0.047

Choice of final restoration	Amalgam	26.7%	27.9%	16.5%	33.3%	24.7%	0.130
	Resin composite	78.5%	78.8%	89.0%	79.3%	76.7%	
	Inlay/onlay	20.3%	9.6%	13.2%	12.6%	14.4%	
	Crown restoration	47.7%	51.9%	52.7%	55.2%	44.5%	
